# NmrLineGuru: Standalone and User-Friendly GUIs for Fast 1D NMR Lineshape Simulation and Analysis of Multi-State Equilibrium Binding Models

**DOI:** 10.1038/s41598-019-52451-8

**Published:** 2019-11-05

**Authors:** Chao Feng, Evgenii L. Kovrigin, Carol Beth Post

**Affiliations:** 1 Department of Medicinal Chemistry and Molecular Pharmacology, Markey Center for Structural Biology, and Purdue Center for Cancer Research, Purdue University, West Lafayette, IN 47907 USA; 2 Magnetic Resonance Research Center, Department of Chemistry and Biochemistry, University of Notre Dame, Notre Dame, IN 46556 USA; 3 Department of Biological Sciences, Purdue University, West Lafayette, IN 47907 USA

**Keywords:** Biophysical chemistry, Bioanalytical chemistry, Software, NMR spectroscopy, Solution-state NMR, Drug discovery

## Abstract

The ability of high-resolution NMR spectroscopy to readout the response of molecular interactions at multiple atomic sites presents a unique capability to define thermodynamic equilibrium constants and kinetic rate constants for complex, multiple-step biological interactions. Nonetheless, the extraction of the relevant equilibrium binding and rate constants requires the appropriate analysis of not only a readout that follows the equilibrium concentrations of typical binding titration curves, but also the lineshapes of NMR spectra. To best take advantage of NMR data for characterizing molecular interactions, we developed NmrLineGuru, a software tool with a user-friendly graphical user interface (GUI) to model two-state, three-state, and four-state binding processes. Application of NmrLineGuru is through stand-alone GUIs, with no dependency on other software and no scripted input. NMR spectra can be fitted or simulated starting with user-specified input parameters and a chosen kinetic model. The ability to both simulate and fit NMR spectra provides the user the opportunity to not only determine the binding parameters that best reproduce the measured NMR spectra for the selected kinetic model, but to also query the possibility that alternative models agree with the data. NmrLineGuru is shown to provide an accurate, quantitative analysis of complex molecular interactions.

## Introduction

NMR is a powerful technique to study molecular structure, dynamics and kinetics without the need for chemical modification. Due to its atom-level resolution, it is particularly useful to study molecular interactions involving multi-state equilibrium and can be used to simultaneously extract thermodynamics and kinetics parameters of each binding step^1–5^. In contrast, the readout of other label-free techniques, such as isothermal titration calorimetry, contains averaged information of multiple binding steps and/or provides estimates of only thermodynamics parameters^6,7^.

To study binding interactions by NMR, a titration experiment is usually performed. At each titration point, an NMR-observable molecule (P) is mixed with its binding partner (L) at a certain ratio and 1D or 2D NMR spectra are recorded. The changes of chemical shifts, peak widths, and peak intensities observed during this process are then used to extract thermodynamics and kinetics parameters.

NMR titration data exhibit different behavior depending on the binding process and exchange regimes set by the relative values of the on/off kinetic rates and chemical shift frequencies of the various molecular species. For the fast-exchange regime, the observed chemical shifts are a weighted average of the two exchanging states and thus thermodynamic parameters can be extracted directly from the normalized chemical shifts as for any titration binding curve^1,8^. For the slow-exchange regime, the peak intensities of exchanging states are proportional to their population and thus can be used to extract thermodynamic parameters^1,6^. However, if the exchange falls in the intermediate regime or involves multiple binding steps or both thermodynamics and kinetics parameters are wanted, a lineshape analysis using the whole peak envelope is necessary.

Although lineshape analysis can extract both thermodynamics and kinetics parameters for multi-state equilibrium binding models and all types of exchange regimes, the calculation steps are more complicated by involving both the equilibrium binding constants and kinetics rate constants. This complication limits the wide application of NMR lineshape analysis method.

Many published works involving NMR lineshape analysis utilized home-written scripts which cannot be easily adopted^2,3^. There are also several published software packages for NMR lineshape analysis which are summarized in Table 1. All the listed NMR lineshape analysis software packages require MATLAB installation, which is a commercial computing environment with expensive licensing fees. In addition, the MATLAB language syntax changes in different versions and older versions (required for older software) are harder to obtain and use as the compatible operating systems become obsolete. In addition, none of the listed software packages is fully based on a graphical user interface (GUI) for both lineshape simulation and fitting; running the script-based simulation or fitting requires a certain level of programming knowledge for MATLAB.

**Table 1:** Summary of existing NMR lineshape software. The shown MATLAB version is known to work with the indicated software but may not be the only version. “?” indicates unknown version. “N/A”: not available in the indicated software.

Name	Dependency	Extra requirements	Simulation functionality	Fitting functionality
TITAN^12^	NMRPipe MATLAB R2016b*	Spec. acqu. parameters 2D lineshape only	Scripts-based Up to 4 states	GUI-based Up to 4 states
IDAP^1,2^	MATLAB R2014a	1D lineshape only	Scripts-based Up to 6 states	Scripts-based Up to 3 states
LineShapeKin Simulation^1^	MATLAB R2011b	1D lineshape only	Scripts-based Up to 6 states	N/A
LineShapeKin^13^	MATLAB R2011b	1D lineshape only	N/A	Scripts-based Up to 2 states
NMRKIN^14^	NMRLab MATLAB?	1D lineshape only	Scripts-based Up to 3 states	Scripts-based Up to 3 states

^*^TITAN currently has standalone version for OS X and Linux, but “some features of TITAN are not available in the standalone application, and are currently only accessible from within MATLAB”.

Based on our previous work in NMR lineshape analysis utilizing multi-state equilibrium models^3^, we developed NmrLineGuru, a standalone and user-friendly NMR lineshape software containing six GUIs for simulating and fitting NMR lineshape with two-, three-, and four-state binding models. These GUIs have no dependency on other software; NmrLineGuru is developed in the MATLAB environment, but does not require separate installation of MATLAB. NmrLineGuru aims to be extremely user-friendly for non-experts and time-saving with the hope of promoting the application of NMR lineshape analysis in research.

## Results and Discussion

### Models and interface

**Figure 1:**
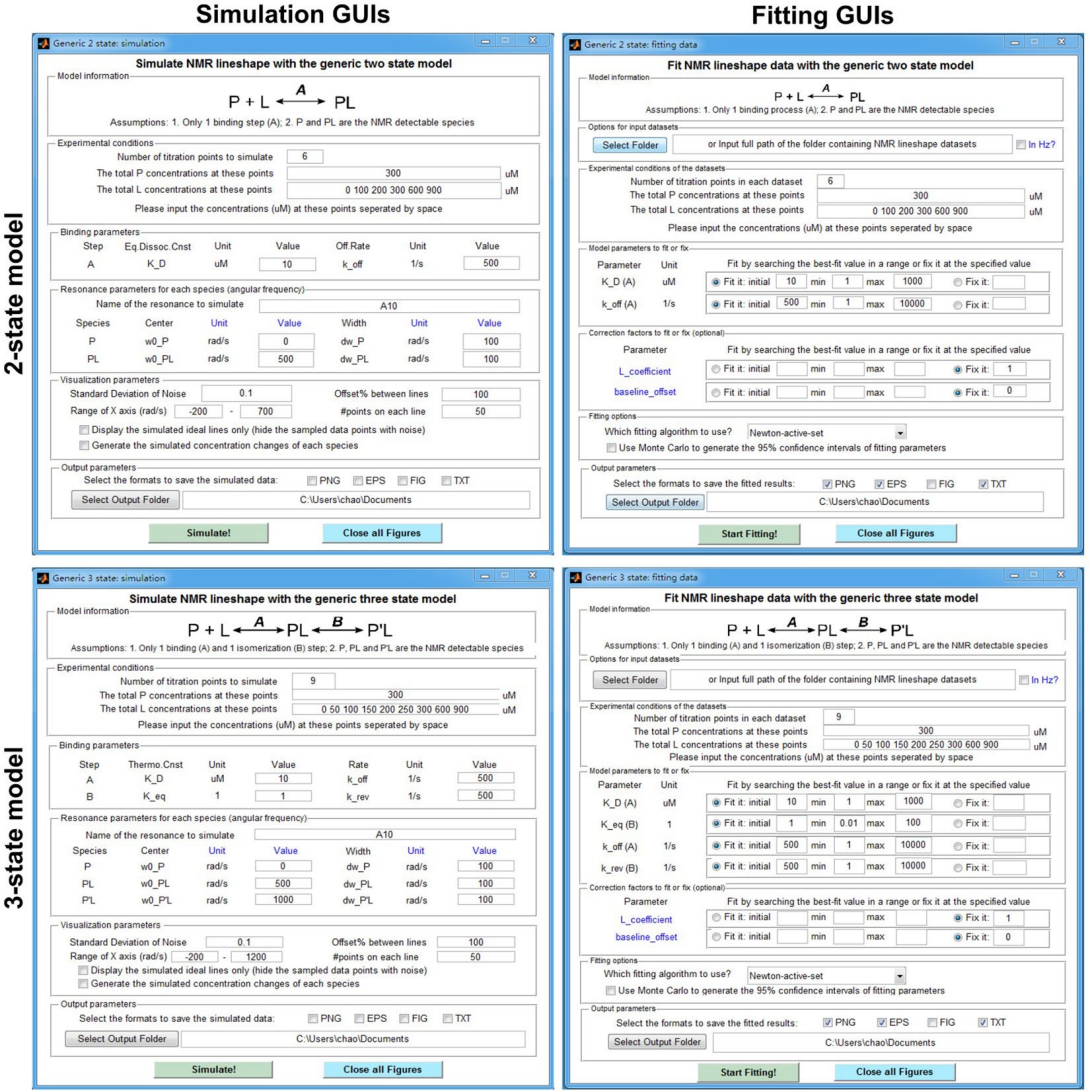
Example lineshape simulation and fitting GUIs. Shown are GUIs for the 2- and 3-state models. GUIs for the 4-state model are shown in Supporting Information Fig. S1. Each GUI is an independent single-window application. Example parameters are filled in and can be used for quick simulation or fitting. User-provided values enable customized simulations or fittings of NMR lineshape data.

NmrLineGuru aims to be extremely friendly for non-experienced users. It currently supports the most commonly used two-, three-, and four-state binding models. For each model, there are two single-window GUI applications, one for simulating and the other for fitting 1D NMR lineshape data. Figure 1 shows screenshots of the 2- and 3-state GUIs as examples (see Supporting Information Fig. S1 for the 4-state GUIs). The input fields on each GUI are grouped and arranged by logical order to facilitate use. Default parameters are filled in by example, and to enable one-click simulation or fitting.

### Overview of the workflow

**Figure 2:**
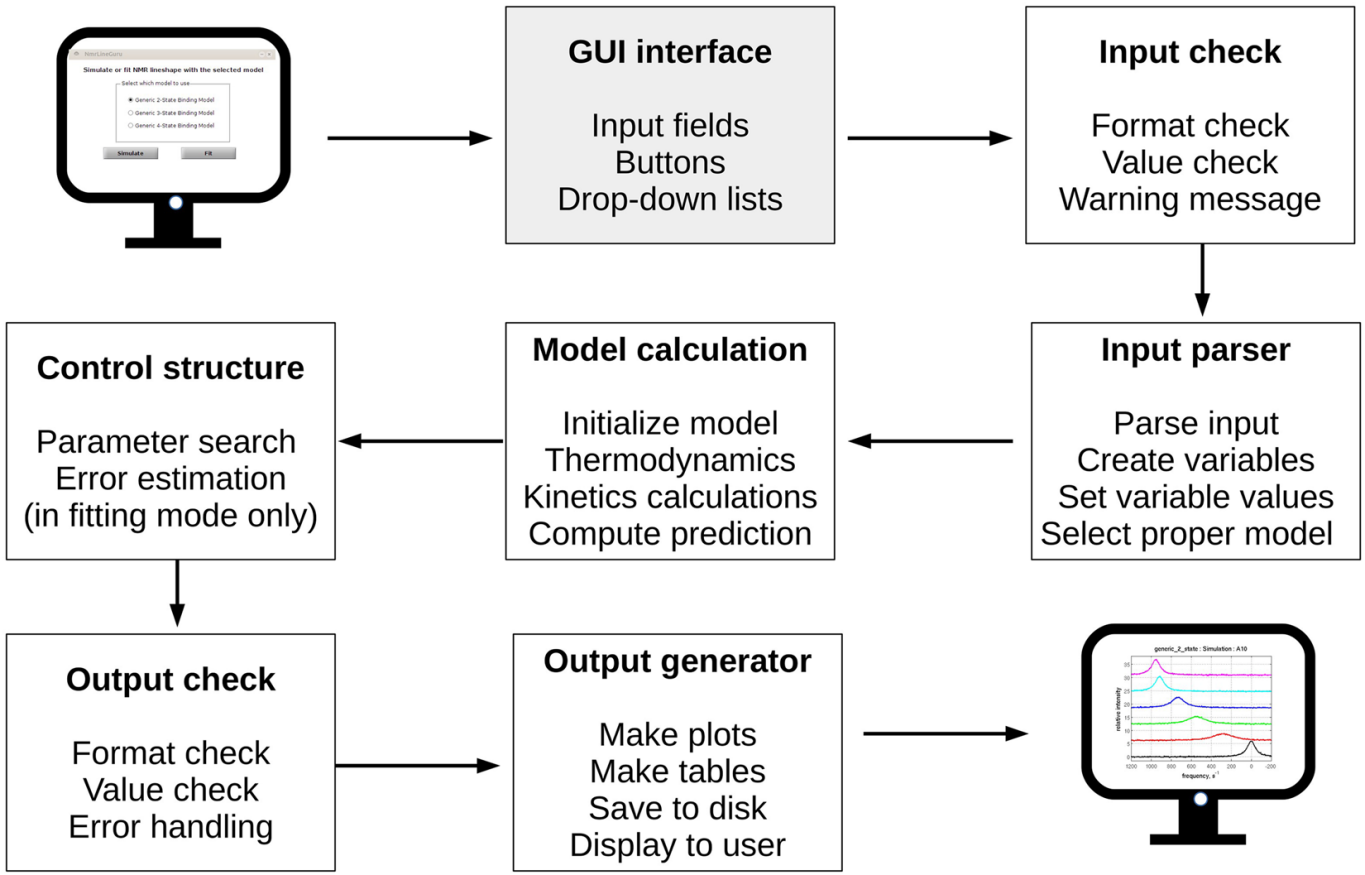
Workflow of NmrLineGuru. Users only need to interact with the graphical user interface (GUI) while all other steps are handled automatically by the software. The results are displayed as on-screen plots and written to the user-specified folder in chosen formats.

The general workflow of NmrLineGuru is shown in Fig. 2. Users are expected to provide only the minimal amount of information in the single-window GUI and the GUI will handle the subsequent steps automatically.

The simulation GUIs take the default or user-provided thermodynamic, kinetic, and resonance parameters to generate 1D NMR lineshape data for the user-specified concentrations of each species in a given titration series according to the selected model. The generated data can contain arbitrary noise and any number of points as requested by the user. The results will be automatically plotted, displayed, and saved in various formats (png, eps, fig, and txt) according to user preference.

The fitting GUIs read in the user-provided lineshape data to search for the best-match thermodynamic and kinetic parameters. The input can be data from the simulation GUIs, experimental data from 1D NMR spectra, or 1D slices from 2D HSQC experiments. The input format (detailed in online tutorials) is simple two-column text files that can be generated or converted from most NMR-spectrum software packages. For the users’ convenience, a plugin for data export is provided and integrated in the NMRFAM^9^ distribution of Sparky (Goddard TD & Kneller DG, University of California, San Francisco), the most popular NMR spectra visualization tool^9^. After reading the lineshape data and initial parameters, the fitting GUIs automatically normalize the input data, perform a Lorentzian fit to estimate resonance- parameters, iteratively search the best-fit dynamic and kinetic parameters within the user-specified range of values, perform Monte Carlo error estimation, and output the results as plots and tables. Automatic global fitting will be performed if multiple datasets are found in the input data directory.

### Example 2-state simulations and fittings

**Figure 3:**
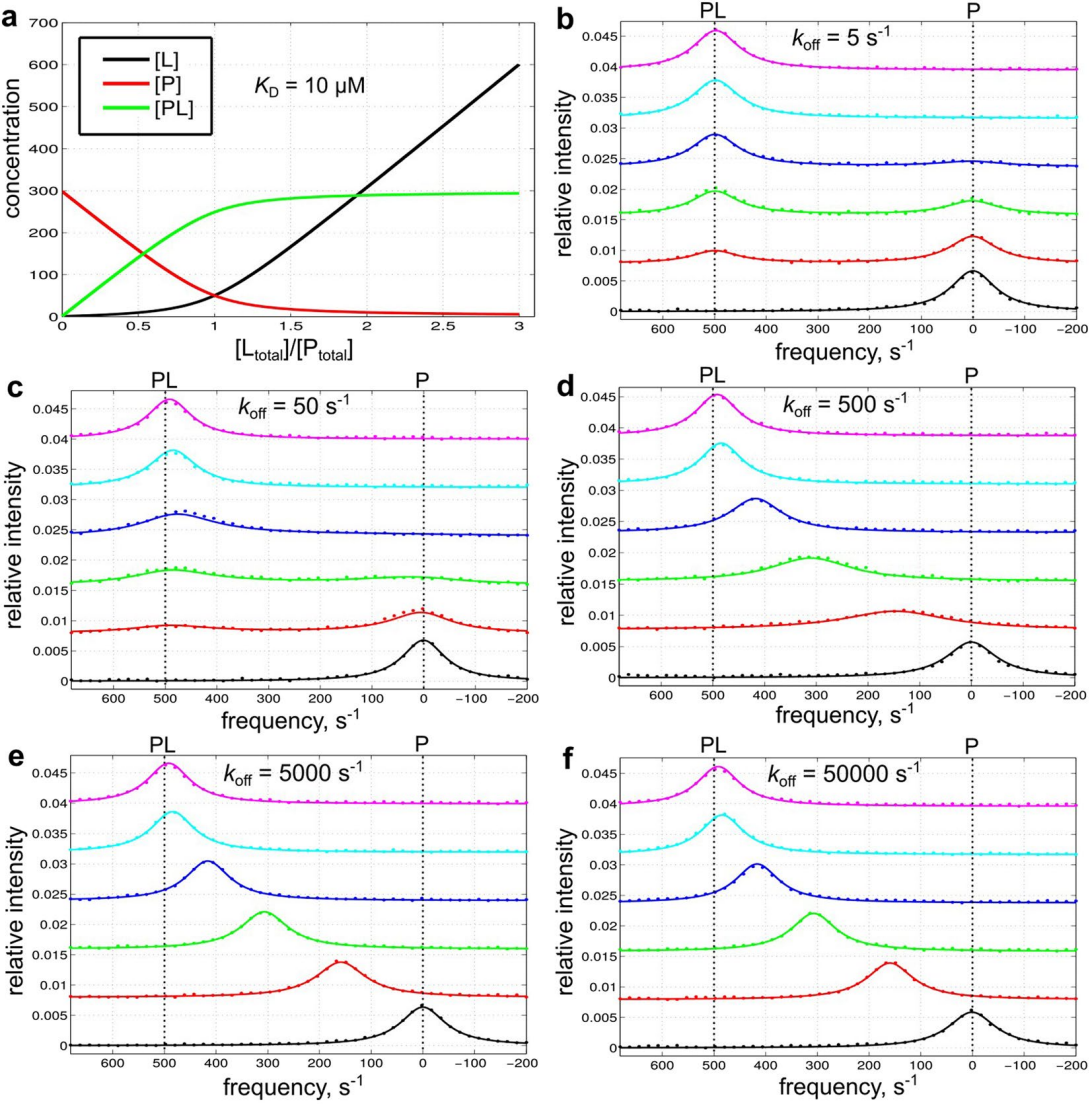
Example 2-state simulations. Simulations use a range of *k*_off_ values and the prefilled default values for other parameters: 300 μM [P_total_]; 0, 100, 200, 300, 600, 900 μM [L_total_]; 10 μM *K*_D_; ω_0_ = 0, 500 s^−1^; line width dω = 100 s^−1^. (**a**) Simulated concentration change of each species as a function of [L_total_]/[P_total_]. (**b**–**f**) Simulated lineshapes with different *k*_off_ values ranging from 0.01–100 × Δω (500 s^−1^). The simulated data (50 data points on each line with noise, signal/noise ≈50) are shown as dots. These data are subsequently fitted with the 2-state fitting GUI and the fitted lineshapes are shown as smooth curves.

The 2-state simulation GUI simulates the single-step binding process (P + L ↔ PL) with arbitrary parameters. Figure 3 shows example 2-state simulations using the prefilled default parameters (300 μM [P_total_]; 0–900 μM [L_total_]; 10 μM *K*_D_; NMR frequencies ω_0_ = 0, 500 s^−1^ for P and PL, respectively; all line widths dω = 100 s^−1^) with different *k*_off_ values ranging from 0.01 to 100 × Δω (5–50000 s^−1^) to highlight the effects of exchange kinetics on NMR lineshape. For a 2-state binding system (free P and bound PL), the exchange rate is *k*_ex_ = *k*_on_ [L] + *k*_off_, which is dominated by *k*_off_ at low ligand concentration. As expected, the generated lineshapes show canonical slow-exchange behavior (Fig. 3b) when *k*_off_ ≪ Δω and fast-exchange behavior (Fig. 3f) when *k*_off_ ≫ Δω. For moderate *k*_off_ values (0.1–10 ×  Δω), the resonances broaden to various extent and shows intermediate-slow or intermediate-fast behavior (Fig. 3c–e).

These simulated 2-state lineshape data (50 data points sampled on each line with random noise, and signal/noise ≈50; a typical signal/noise level of 50–100 was seen in our NMR titration studies, such as the 29 kD Syk tSH2 constructs^3,7^) are fitted with the 2-state fitting GUI (Fig. 3 and Table 2). In all cases, the GUI correctly determines the *K*_D_ value. *k*_off_ values are determined for the range of 0.1–10 ×  Δω, and cannot be determined for values ≥ 100 × Δω, which is above the fast-exchange limit (i.e. spectrum appearance becomes insensitive to the *k*_off_ values above the limit).

**Table 2:** Summary of example 2-state fittings. The input data are from 2-state simulations shown in Fig. 3 as indicated, containing noise (signal/noise ≈50) and 50 data points on each line. The data are fitted with the 2-state fitting GUI. The parameter searching range is [1 nM, 1 mM] for *K*_D_ and [0.1 s^-1^, 100000 s^−1^] for *k*_off_. The fitted parameter values are shown with 95% confidence intervals determined by Monte Carlo resampling. UB: not determined due to reaching the upper boundary.

Figure	True parameters		2-state fitting	
	*K*_D_ (μM)	*k*_off_ (s^−1^)	*K*_D_ (μM)	*k*_off_ (s^−1^)
3b	10	5	11 ± 2	5.2 ± 0.7
3c	10	50	11 ± 1	45 ± 3
3d	10	500	10 ± 1	580 ± 40
3e	10	5000	10 ± 1	6300 ± 1500
3f	10	50000	10 ± 1	UB

### Example 3-state simulations and fittings

**Figure 4:**
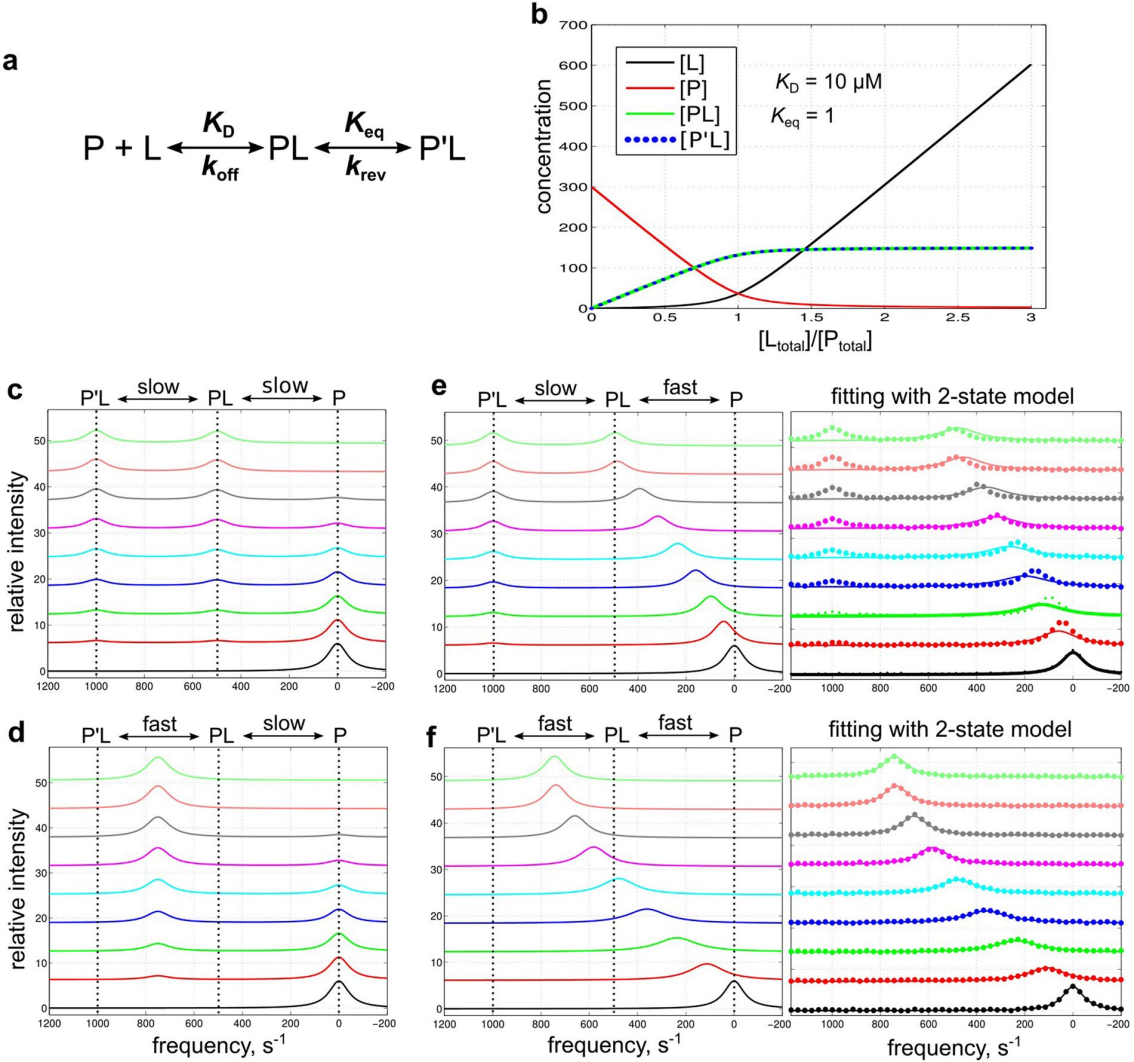
Example 3-state simulations. Simulations use a range of kinetic rates and the prefilled default values for other parameters: 300 μM [P_total_]; 0, 50, 100, 150, 200, 250, 300, 600, 900 μM [L_total_]; 10 μM *K*_D_; 1 *K*_eq_; ω_0_ = 0, 500, 1000 s^−1^; line width dω = 100 s^−1^. (**a**) The 3-state binding model. (**b**) Simulated concentration change of each species as a function of [L_total_]/[P_total_]. (**c**–**f**) Simulated lineshape with different *k*_off_/*k*_rev_ values (slow: 5 s^−1^; fast: 5000 s^−1^). Fifty data points on each simulated line are sampled with noisy (signal/noise ≈50) and subsequently fitted with the 3- or 2-state fitting GUI. For panels e and f, the sampled data points and fitted curves with the 2-state GUI are shown on the right as examples. See Supporting Information Figs S2 and S3 for all fittings.

The 3-state simulation GUI can simulate any [L]-dependent binding followed by a [L]-independent step such as ligand isomerization or ligand-induced protein conformational change (Fig. 4a). Figure 4 displays example 3-state simulations (300 μM [P_total_]; 0–900 μM [L_total_]; 10 μM *K*_D_; 1 K_eq_; NMR frequencies ω_0_ = 0, 500, 1000 s^−1^ for P, PL and P′L, respectively; all line widths dω = 100 s^−1^) for fast or slow binding coupled with fast or slow isomerization (see Table 3 for *k*_off_ and *k*_rev_ values). For simplification, intermediate exchange is not illustrated for either step. For this 3-state binding system (free P, bound PL and isomerized P′L), the isomerization step lacks dependency on ligand concentration so species PL and P′L form at a constant ratio defined by the equilibrium constant *K*_eq_ = [PL]/[P′L]. Coupled with the [L]-dependent exchange between P and PL, this system has interesting behavior. When the isomerization step is slow exchange (Fig. 3c,e), PL and P′L resonances are separate peaks; the system can appear like 2-state exchange between P and PL or P′L depending on *K*_eq_. When the isomerization step is fast exchange (Fig. 3d,f), PL and P′L resonances show up as one peak; the system looks like 2-state exchange between P and the PL/P′L-degenerate peak. The behavior therefore can be deceptive and careful inspection of the spectra is needed when investigating the detailed binding mechanism^1^.

**Table 3:** Summary of example 3-state fittings. The input data are from 3-state simulations shown in Fig. 4 as indicated, containing noise (signal/noise ≈50) and 50 data points on each line. The data are fitted with the 3- or 2-state fitting GUI. The parameter searching range is [1 µM, 1 mM] for *K*_D_, [0.01, 100] for *K*_eq_, and [1, 10000] for *k*_off_/*k*_rev_. The fitted parameter values are shown with 95% confidence intervals determined by Monte Carlo resampling. LB: not determined due to reaching lower boundary. SV: confidence interval not determined due to small variation of the fitted parameter from Monte Carlo runs.

Figure	True parameters				3-state fitting				2-state fitting	
	*K*_D_ (μM)	*K*_eq_	*k*_off_ (s^−1^)	*k*_rev_ (s^−1^)	*K*_D_ (μM)	*K*_eq_	*k*_off_ (s^−1^)	*k*_rev_ (s^−1^)	*K*_D_ (μM)	*k*_off_ (s^−1^)
4c	10	1	5	5	16 ± 3	0.98 ± 0.03	2.8 ± 1.5	5.5 ± 1.2	44 ± 4	2.0 ± 1.4
4d	10	1	5	5000	11 ± 2	1.0 ± 0.1	2.6 ± 1.5	3100 ± SV	4.5 ± 0.8	LB
4e	10	1	5000	5	9.7 ± 0.5	0.99 ± 0.02	4500 ± SV	9.3 ± 1.2	28 ± 1	750 ± 50
4 f	10	1	5000	5000	10 ± 1	0.99 ± 0.01	3300 ± SV	7100 ± SV	4.4 ± 0.2	1400 ± 100

The combined fitting and simulation capability of NmrLineGuru enables facile consideration of different binding models. The simulated 3-state lineshape data (50 data points on each line with noise, signal/noise ≈50) were fitted with the 2- and 3-state fitting GUIs. The results are summarized in Table 3. As required, the 3-state model fits well with all the lineshape data (Supporting Information Fig. S2), and gives correct thermodynamic and kinetic parameters within 95% confidence intervals for most values. The difference in *K*_D_ estimated from resonance in Fig. 4c and the actual value is less than a factor of two, which is typically considered to be within experimental error in practice. Interestingly, the 2-state model agrees well with the lineshape data when the isomerization is in fast exchange (Fig. 4d,f and Supporting Information Fig. S3). When both steps are in slow exchange, and the resonances for P and PL are selected for the 2-state fitting, ignoring the P′L resonance, the lineshapes are well fitted (Fig. 4c and Supporting Information Fig. S3). But for the case where ligand binding is in fast exchange and isomerization is slow (Fig. 4e), fitting is poor even though the P′L resonance is ignored and deviations in fitting the NMR lineshapes are sufficient to suspect an incorrect model. Nevertheless, even for the cases where the lineshapes are well fitted, the *K*_D_ and *k*_off_ values are inaccurate, especially when both the binding and isomerization steps are in slow exchange (Table 3). In part, the apparent good fit of the lineshapes is due to the normalization step imposed in the fitting algorithm.

### Example 4-state simulations and fittings

**Figure 5:**
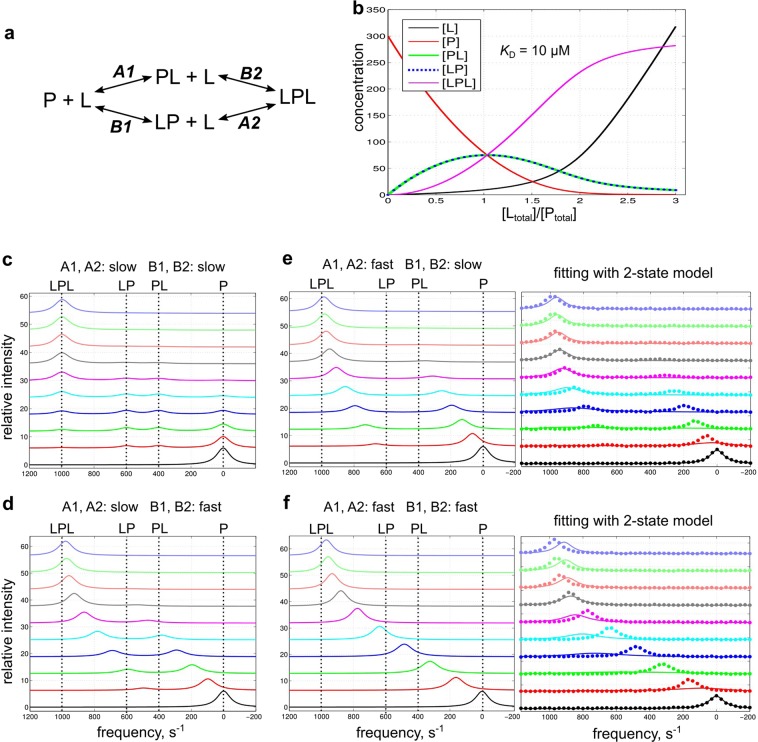
Example 4-state simulations. Simulations use a range of kinetic rates and the prefilled default values for other parameters: 300 μM [P_total_]; 0, 100, 200, 300, 400, 500, 600, 700, 800, 900 μM [L_total_]; *K*_D_ 10 μM for the four binding steps; ω_0_ = 0, 400, 600, 1000 s^−1^; line width dω = 100 s^−1^. (**a**) The 4-state binding model. (**b**) Simulated concentration change of each species as a function of [L_total_]/[P_total_]. (**c**–**f**) Simulated lineshape with different *k*_off_ values (slow: 5 s^−1^; fast: 5000 s^−1^) for each binding step. Fifty data points on each simulated line are sampled with noise (signal/noise ≈50) and subsequently fitted with the 4- or 2-state fitting GUI. For panel (**e**,**f**), the sampled data points and fitted curves with the 2-state GUI are shown on the right as examples. See Supporting Information Figs S4 and S5 for all fittings.

The 4-state simulation GUI can simulate any system with two independent or coupled binding sites and arbitrary binding affinities (Fig. 5a). Figure 5 shows example 4-state simulations for two independent binding sites with similar binding affinities (300 μM [P_total_]; 0–900 μM [L_total_]; 10 μM *K*_D_ for all binding steps; NMR frequencies ω_0_ = 0, 400, 600, 1000 s^−1^ for P, PL, LP and LPL, respectively; all line widths dω = 100 s^−1^), and disparate kinetic rate constants (see Table 4). Lineshape simulations are only shown for the limiting conditions of fast or slow exchange. When binding to both sites is in slow exchange, the NMR lineshape shows four resonances for the free, two singly ligated (LP and PL) and doubly ligated (LPL) forms of the protein (Fig. 5c), with the peak intensity for P gradually decreasing, that for LP and PL increasing and then decreasing, and that for LPL gradually increasing, which directly reflects the population change shown in Fig. 5b. When one of the two sites is in fast exchange, the NMR lineshape is dominated by the rapid kinetics for binding either free protein or protein ligated at the alternate site, and the spectrum looks like two fast-exchange binding processes in that the line frequency appears to follow the equilibrium concentration; however, the peak intensity for free P decreases and that for the ligated form increases (Fig. 5d,e). When both sites are in fast exchange, the NMR lineshape is collapsed into a single resonance at all titration points and looks like a 2-state binding system (Fig. 5f).

**Table 4:** Summary of example 4-state fittings. The input data are from 4-state simulations shown in Fig. 5 as indicated, containing noise (signal/noise ≈50) and 50 data points on each line. The data are fitted with the 4- or 2-state fitting GUI. The parameter searching range is [1 µM, 1 mM] for all *K*_D_ values and [1 s^−1^, 10000 s^−1^] for all *k*_off_ values. The fitted parameter values are shown with 95% confidence intervals determined by Monte Carlo resampling. UB: not determined due to reaching upper boundary. SV: confidence interval not determined due to small variation of the fitted parameter from Monte Carlo runs.

Figure	True parameters			4-state fitting			2-state fitting	
	*K*_D_ (μM) A1 = A2 = B1 = B2	*k*_off_ (s^−1^) A1 = A2	*k*_off_ (s^−1^) B1 = B2	*K*_D_ (μM) A1 = A2 = B1 = B2	*k*_off_ (s^−1^) A1 = A2	*k*_off_ (s^−1^) B1 = B2	*K*_D_ (μM)	*k*_off_ (s^−1^)
5c	10	5	5	16 ± 2	5.0 ± 1.3	5.6 ± 1.4	70 ± 3	18 ± 1
5d	10	5	5000	10 ± 1	6.7 ± 0.7	UB	23 ± 1	300 ± 10
5e	10	5000	5	10 ± 1	2600 ± SV	6.7 ± 0.7	15 ± 1	200 ± 10
5 f	10	5000	5000	10 ± 1	2077 ± SV	UB	39 ± 1	380 ± 20

These simulated 4-state lineshape data (50 data points on each line with noise, signal/noise ≈50) were fitted with the 2- or 4-state fitting GUI. The 2-state model cannot fit any of the lineshape data, not even those from Fig. 5f which look like 2-state exchange (Fig. 5e,f; Supporting Information Fig. S4). In contrast, the 4-state model fits well with all lineshape data (Supporting Information Fig. S5) and gives correct values for all *K*_D_ parameters and most of the *k*_off_ parameters (Table 4). There are two *k*_off_ parameters which cannot be determined from the 4-state fitting due to reaching the fast-exchange limit (“UB” in Table 4).

### Experimental lineshape data for Syk tSH2 binding with N-IHP

The GUIs were used to examine data reported for the interaction between Syk tandem SH2 domain and tyrosyl phosphorylated peptides^3^. In previous work, the necessity and power of using NMR lineshape analysis to effectively extract dynamic and kinetic information was recognized^3^. NmrLineGuru is demonstrated here to analyze data on this interaction.

**Figure 6:**
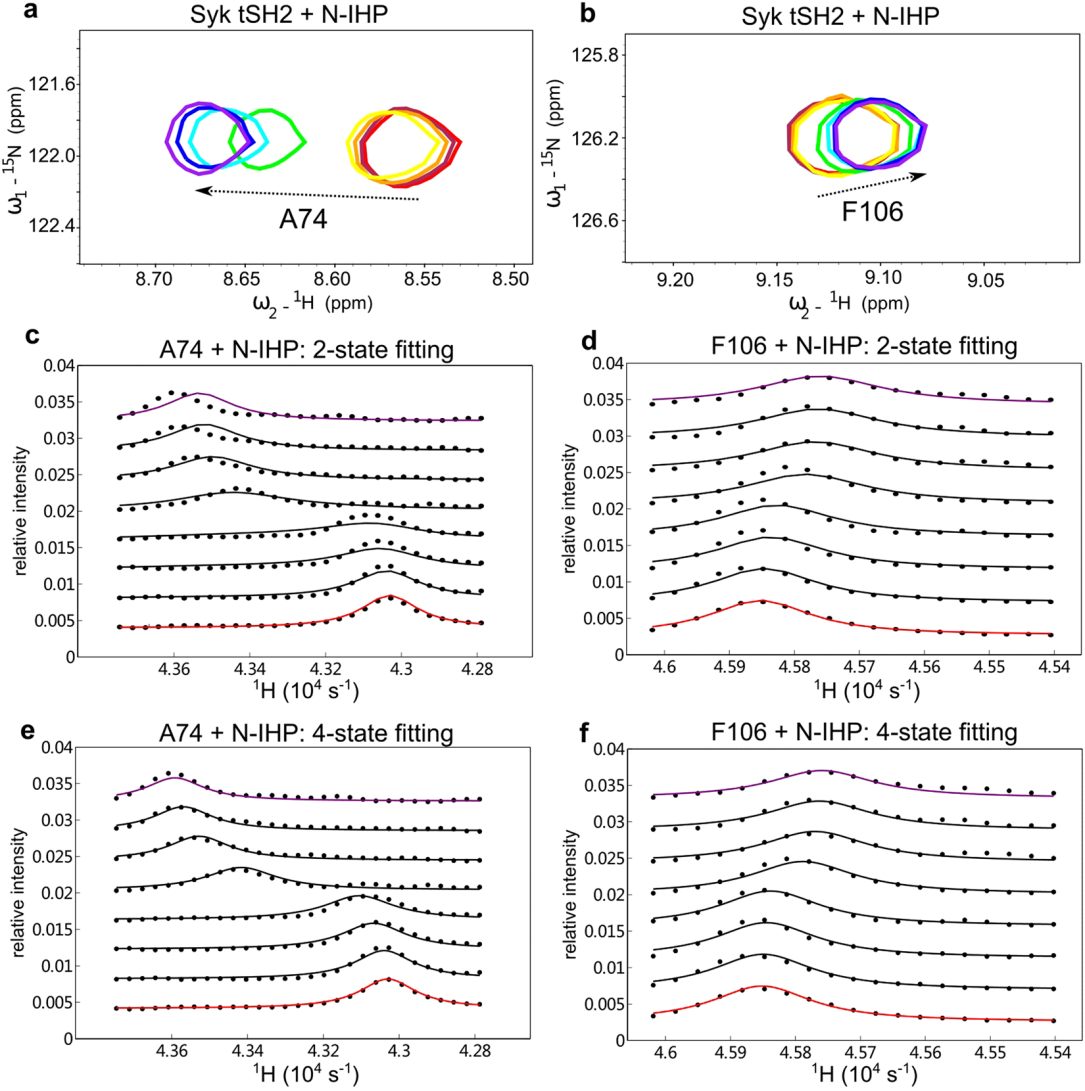
Example NMR ^15^N-^1^H HSQC chemical shift titration data and comparison of lineshape fitting with the 2-state and 4-state models. (**a**,**b**) Overlaid HSQC spectra for the unphosphorylated Syk tSH2 (~0.3 mM) titration with the peptide N-IHP [Ac-PD(pY)EPIRKG-NH_2_]. Increasing [N-IHP]/[tSH2] ratios correspond to color changes: 0 (red), 0.2 (maroon), 0.6 (orange), 1.0 (yellow), 5.0 (green), 10.1 (cyan), 15.1 (blue), and 20.1 (purple). Zoomed regions with A74 and F106 are shown as examples. The arrows indicate the shift of peaks during the titration process. (**c**–**f**) Lineshape analysis for residues A74 and F106 in tSH2 plus N-IHP, with either the conventional 2-state model or the 4-state model. Circles: raw lineshape data. Lines: predicted lineshape with the indicated model (Red: the first titration point with no ligand. Purple: the last titration point with high concentration of ligand). The 4-state model fits well with the NMR data while the 2-state model does not.

The Syk tandem SH2 domain fragment (tSH2) contains two SH2 domains and either domain can bind to a phosphotyrosine peptide, N-IHP [Ac-PD(pY)EPIRKG-NH_2_], with a sequence derived from the CD3ε chain of the T cell receptor. tSH2 was titrated with N-IHP and the binding process was monitored by ^15^N-^1^H HSQC experiments (Fig. 6a,b). Although the lineshapes look like 2-state exchange, it is demonstrated that the 2-state model cannot fit the data (Fig. 6c,d). The lineshapes of resonances from both domains (proton-dimension slices for G32, L37, H61, A74, F106, D175, G184, and G210; nitrogen-dimension slices for L52, Y73, L192, C205, and S244) are then globally fitted with the 4-state model assuming two independent binding sites and the 4-state model fits the data well (Fig. 6e,f). The fitted equilibrium dissociation constants are 730 ± 20 and 69 ± 4 µM and the fitted off rates are 1800 ± 300 and 380 ± 20 s^−1^ (mean ± SD of fitted values from two independent experiments), for the N- and C-terminal SH2 domains binding N-IHP, respectively.

### Comparison of the fitted parameters by IDAP, TITAN, and NmrLineGuru

**Figure 7:**
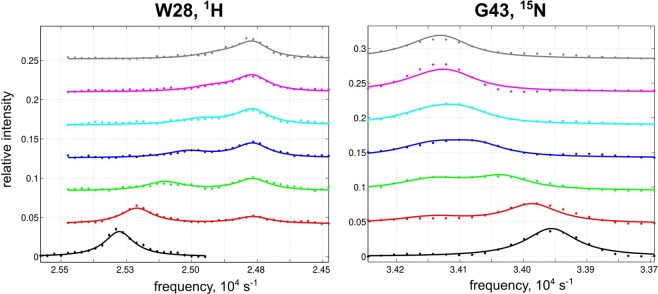
Example NMR ^15^N-^1^H HSQC 1D spectral slice data from E22A CsnN174 chitosanase (~0.08 mM) titration^10^ with the chitosan hexamer substrate (GlcN)_6_ and the lineshape fitting using NmrLineGuru. Increasing ligand/protein molar ratios correspond to color changes: 0 (black), 0.5 (red), 1 (green), 1.5 (blue), 2 (cyan), 3 (magenta), and 5 (gray). Lineshape analysis and global fitting were performed with the 3-state fitting GUI in NmrLineGuru for the following resonances: proton dimension for W28, G39, G153, and T157; nitrogen dimension for G43 and T157. Circles: raw lineshape data. Lines: predicted lineshape from global fitting with NmrLineGuru.

The experimental lineshape data^10^ for E22A CsnN174 chitosanase binding with the chitosan hexamer substrate (GlcN)_6_ were used to compare the fitting quality of NmrLineGuru to two other packages, IDAP^1,2^ and TITAN^12^. Data from multiple peaks were globally fitted in all three cases. Example lineshapes and fittings by NmrLineGuru are shown in Fig. 7 and the fitted parameters are summarized in Table 5 (fitted values with IDAP and TITAN are from reference^10^).

**Table 5:** Comparison of the fitted parameters by IDAP, TITAN, and NmrLineGuru for the E22A CsnN174 chitosanase titration data^10^. The input data for IDAP and NmrLineGuru are the six 1D spectral slices described in Fig. 7 while TITAN uses nine amide NH 2D peaks^10^ (residues N23, W28, A30, G39, G43, G47, G153, T157, and D232). The fitted values for IDAP and TITAN are from reference^10^. The fitted parameter values for NmrLineGuru are shown with 95% confidence intervals determined by Monte Carlo resampling. UB: not determined due to reaching the upper boundary (50000 s^−1^).

Software	Fitted parameters with the 3-state model			
	*K*_D_ (μM)	*K*_eq_	*k*_off_ (s^−1^)	*k*_rev_ (s^−1^)
IDAP	42	0.26	30000	9
TITAN	36 ± 2	0.32	17000 ± 12000	3.6 ± 0.9
NmrLineGuru	29 ± 2	0.36 ± 0.01	UB	8.7 ± 0.9

The lineshape behavior shown in Fig. 7, especially that of W28, resembles the simulation in Fig. 4e whereby one peak shifts and decreases intensity, and another peak gradually appears. This behavior indicates that the underlying mechanism is fast ligand binding coupled with slow isomerization, consistent with the reported analysis^10^. Global fitting of the selected 1D spectral slices by NmrLineGuru gives similar thermodynamic and kinetic parameters to those from IDAP and TITAN; the differences shown in Table 5 are considered negligible. The off rate of the ligand binding step is too fast to be accurately determined, which for NmrLineGuru is reported as beyond fast-exchange limit.

## Conclusion

We describe six standalone and user-friendly GUIs for both simulating and fitting 1D NMR lineshape data using the common 2-, 3-, or 4-state binding models. Accuracy in fitting the NMR data was demonstrated with simulated and experimental data, including global fitting of multiple peak lineshapes. These GUIs require the minimal amount of user input and handle most of the workflow in an automatic way. Aiming for non-experienced users, these GUIs can help to promote the wide use of the NMR lineshape analysis method, which is powerful and unique in studying dynamics and kinetics for multi-state binding systems.

## Methods

### Code development

The GUIs are developed in MATLAB R2014a (The MathWorks, Inc.) and compiled into standalone applications for both Windows and Linux. The low-level 1D NMR lineshape data I/O APIs (including the Sparky plugin) are from IDAP^1,2^ (Integrative Data Analysis Platform, http://lineshapekin.net).

### NMR lineshape for an exchange system

The NMR transverse magnetization for an equilibrium system of *N* spins in chemical exchange is generally described by the matrix form of the Bloch-McConnell equations^2,11^. The spectrum intensity at angular frequency ω is given by the sum of real components of the following complex vector **S**:

1 $${\bf{S}}={{\boldsymbol{\Omega }}}^{-1}{\bf{P}}$$

where **P** is a complex vector of length *N* and **Ω** is a *N* × *N* complex matrix. The elements of **P** are the relative spin populations, *p*_*i*_, defined by the kinetic rate constants ${k}_{ij}^{\ast }$

2 $${\rm{spin}}\,i\,\mathop{\leftarrow }\limits_{{k}_{ji}^{\ast }}\,\mathop{\to }\limits^{{k}_{ij}^{\ast }}{\rm{spin}}\,j$$

**Ω** is given by

3 $${\boldsymbol{\Omega }}={\bf{M}}-{\bf{K}}$$

**M** is a diagonal matrix with the following element *M*_*ii*_ for spin *i*

4 $${M}_{ii}={R}_{2}^{i}+i2\pi (\omega -{\omega }_{0}^{i})$$

where ${R}_{2}^{i}$ and ${\omega }_{0}^{i}$ are the transverse relaxation rate and intrinsic resonance frequency of spin *i*. ${R}_{2}^{i}$ and ${\omega }_{0}^{i}$ are provided by user input for simulations, or obtained by fitting the given resonance peaks to a Lorentzian function (assuming the peak centers at *ω*_0_ with a line width 2*R*_2_), or searched as fitting parameters during fitting procedures. **K** is the *N* × *N* exchange matrix with the following elements from kinetic rate constants:

5 $${K}_{ij}={k}_{ji}^{\ast }(i\ne j)$$

6 $${K}_{ii}=-\mathop{\sum }\limits_{j\ne i}^{N}\,{k}_{ji}^{\ast }$$

### Theory for 2-state binding and lineshape analysis

The 2-state binding model contains only one binding step between P and L to form PL, assuming P and PL are the NMR-detectable species. The system contains one equilibrium dissociation constant, *K*_D_:

7 $${K}_{{\rm{D}}}=\frac{[{\rm{P}}][{\rm{L}}]}{[{\rm{PL}}]}$$

The total concentration of protein and ligand of each titration point is:

8 $$[{{\rm{P}}}_{{\rm{total}}}]=[{\rm{P}}]+[{\rm{PL}}]$$

9 $$[{{\rm{L}}}_{{\rm{total}}}]=[{\rm{L}}]+[{\rm{PL}}]$$

From Eqs (7–9), the relationship between [L], [P_total_], [L_total_], and *K*_D_ can be solved as:

10 $${[{\rm{L}}]}^{2}+([{{\rm{P}}}_{{\rm{total}}}]-[{{\rm{L}}}_{{\rm{total}}}]+{K}_{{\rm{D}}})[{\rm{L}}]-{K}_{{\rm{D}}}[{{\rm{L}}}_{{\rm{total}}}]=0$$

With given values of [P_total_], [L_total_], and varying values for *K*_D_, the value of [L] is solved analytically. Once [L] is known, the concentrations of other species in the system are determined based on Eqs (7–9):

11 $$[{\rm{P}}]=\frac{{K}_{{\rm{D}}}[{{\rm{P}}}_{{\rm{total}}}]}{[{\rm{L}}]+{K}_{{\rm{D}}}}$$

12 $$[{\rm{PL}}]=\frac{[{\rm{L}}][{\rm{P}}]}{{K}_{{\rm{D}}}}$$

The NMR lineshape calculation requires an additional parameter to describe the 2-state system, the off rate *k*_off_. The corresponding on rate is:

13 $${k}_{{\rm{on}}}=\frac{{k}_{{\rm{off}}}}{{K}_{{\rm{D}}}}$$

The predicted lineshape for a nucleus at a given titration point is described in Eqs (1–6). The following matrices are now

14 $${\bf{P}}=[\begin{array}{c}{[{\rm{P}}]}\\{[{\rm{PL}}]}\end{array}]$$

15 $${\bf{K}}=[\begin{array}{cc}-{k}_{{\rm{on}}}[{\rm{L}}] & {k}_{{\rm{off}}}\\ {k}_{{\rm{on}}}[{\rm{L}}] & -{k}_{{\rm{off}}}\end{array}]$$

### Theory for the 3- state binding and lineshape

The 3-state binding model (Fig. 4a) contains an inter-molecular binding step between the free P and L to form PL, and an intra-molecular (concentration independent) isomerization step for PL to form P′L. These two steps are described by the equilibrium dissociation constant *K*_D_ and equilibrium isomerization constant *K*_eq_:

16 $${K}_{{\rm{D}}}=\frac{[{\rm{P}}][{\rm{L}}]}{[{\rm{PL}}]}$$

17 $${K}_{{\rm{eq}}}=\frac{[{\rm{PL}}]}{[{\rm{P}}^{\prime} {\rm{L}}]}$$

The total concentration of protein and ligand of each titration point is expressed as

18 $$[{{\rm{P}}}_{{\rm{total}}}]=[{\rm{P}}]+[{\rm{PL}}]+[{\rm{P}}^{\prime} {\rm{L}}]$$

19 $$[{{\rm{L}}}_{{\rm{total}}}]=[{\rm{L}}]+[{\rm{PL}}]+[{\rm{P}}^{\prime} {\rm{L}}]$$

From Eqs (16–19), the relationship between [L], [P_total_], [L_total_], *K*_D_, and *K*_eq_ is

20 $$({K}_{{\rm{eq}}}+1){[{\rm{L}}]}^{2}+(({K}_{{\rm{eq}}}+1)[{{\rm{P}}}_{{\rm{total}}}]-({K}_{{\rm{eq}}}+1)[{{\rm{L}}}_{{\rm{total}}}]+{K}_{{\rm{D}}}{K}_{{\rm{eq}}})[{\rm{L}}]-{K}_{{\rm{D}}}{K}_{{\rm{eq}}}[{{\rm{L}}}_{{\rm{total}}}]=0$$

With given values of [P_total_], [L_total_], *K*_D_, *K*_eq_, the value of [L] can be solved analytically. Once [L] is known, the concentrations of other species in the system could be determined as follows based on Eqs (16–19):

21 $$[{\rm{P}}]=\frac{{K}_{{\rm{D}}}\,{K}_{{\rm{eq}}}\,[{{\rm{P}}}_{\mathrm{total}}]}{({K}_{{\rm{eq}}}+1)[{\rm{L}}]+{K}_{{\rm{D}}}\,{K}_{{\rm{eq}}}}$$

22 $$[{\rm{PL}}]=\frac{[{\rm{P}}][{\rm{L}}]}{{K}_{{\rm{D}}}}$$

23 $$[{\rm{P}}^{\prime} {\rm{L}}]=\frac{[{\rm{PL}}]}{{K}_{{\rm{eq}}}}$$

The lineshape analysis requires two more parameters to describe the 3-state system: the off rate for the binding process (*k*_*off*_) and the reverse rate for the isomerization process (*k*_*rev*_). The corresponding on rate and forward rate are:

24 $${k}_{{\rm{on}}}=\frac{{k}_{{\rm{off}}}}{{K}_{{\rm{D}}}}$$

25 $${k}_{{\rm{fwd}}}=\frac{{k}_{{\rm{rev}}}}{{K}_{{\rm{eq}}}}$$

The predicted lineshape for a nucleus at a given titration point is described in Eqs (1–6). The following matrices are now

26 $${\bf{P}}=[\begin{array}{c}{[{\rm{P}}]}\\{[{\rm{PL}}]}\\{[{\rm{P}}^{\prime} {\rm{L}}]}\end{array}]$$

27 $${\bf{K}}=[\begin{array}{ccc}-{K}_{{\rm{on}}}[{\rm{L}}] & {k}_{{\rm{off}}} & 0\\ {K}_{{\rm{on}}}[{\rm{L}}] & -{k}_{{\rm{off}}}-{k}_{{\rm{fwd}}} & {k}_{{\rm{rev}}}\\ 0 & {k}_{{\rm{fwd}}} & -{k}_{{\rm{rev}}}\end{array}]$$

### Theory for the 4- state binding and lineshape

The 4-state binding model (Fig. 5a) contains four binding steps described by four equilibrium dissociation constants:

28 $${K}_{{\rm{D}}}^{{\rm{A}}1}=\frac{[{\rm{P}}][{\rm{L}}]}{[{\rm{PL}}]}$$

29 $${K}_{{\rm{D}}}^{{\rm{B}}1}=\frac{[{\rm{P}}][{\rm{L}}]}{[{\rm{LP}}]}$$

30 $${K}_{{\rm{D}}}^{{\rm{A}}2}=\frac{[{\rm{LP}}][{\rm{L}}]}{[{\rm{LPL}}]}$$

31 $${K}_{{\rm{D}}}^{{\rm{B}}2}=\frac{{K}_{{\rm{D}}}^{{\rm{B}}1}{K}_{{\rm{D}}}^{{\rm{A}}2}}{{K}_{{\rm{D}}}^{{\rm{A}}1}}$$

Note that only the first three equilibrium dissociation constants are independent parameters while the fourth becomes a function of the first three. The total concentration of protein and ligand of each titration point is expressed as

32 $$[{{\rm{P}}}_{{\rm{total}}}]=[{\rm{P}}]+[{\rm{PL}}]+[{\rm{LP}}]+[{\rm{LPL}}]$$

33 $$[{{\rm{L}}}_{{\rm{total}}}]=[{\rm{L}}]+[{\rm{PL}}]+[{\rm{LP}}]+2[{\rm{LPL}}]$$

From Eqs (28–33), the relationship between [L], [P_total_], [L_total_], ${K}_{{\rm{D}}}^{{\rm{A}}1}$, ${K}_{{\rm{D}}}^{{\rm{B}}1}$ and ${K}_{{\rm{D}}}^{{\rm{A}}2}$ is

34 $$\begin{array}{c}{K}_{{\rm{D}}}^{{\rm{A}}1}\,{[{\rm{L}}]}^{3}+(2{K}_{{\rm{D}}}^{{\rm{A}}1}[{{\rm{P}}}_{{\rm{total}}}]-{K}_{{\rm{D}}}^{{\rm{A}}1}[{{\rm{L}}}_{{\rm{total}}}]+{K}_{{\rm{D}}}^{{\rm{A}}2}{K}_{{\rm{D}}}^{{\rm{B}}1}+{K}_{{\rm{D}}}^{{\rm{A}}1}{K}_{{\rm{D}}}^{{\rm{A}}2}){[{\rm{L}}]}^{2}+(({K}_{{\rm{D}}}^{{\rm{A}}2}{K}_{{\rm{D}}}^{{\rm{B}}1}+{K}_{{\rm{D}}}^{{\rm{A}}1}{K}_{{\rm{D}}}^{{\rm{A}}2})[{{\rm{P}}}_{{\rm{total}}}]\\ -({K}_{{\rm{D}}}^{{\rm{A}}2}{K}_{{\rm{D}}}^{{\rm{B}}1}+{K}_{{\rm{D}}}^{{\rm{A}}1}{K}_{{\rm{D}}}^{{\rm{A}}2})[{{\rm{L}}}_{{\rm{total}}}]+{K}_{{\rm{D}}}^{{\rm{A}}1}{K}_{{\rm{D}}}^{{\rm{A}}2}{K}_{{\rm{D}}}^{{\rm{B}}1})[{\rm{L}}]-{K}_{{\rm{D}}}^{{\rm{A}}1}{K}_{{\rm{D}}}^{{\rm{A}}2}{K}_{{\rm{D}}}^{{\rm{B}}1}[{{\rm{L}}}_{{\rm{total}}}]=0\end{array}$$

With given values of [P_total_], [L_total_], ${K}_{{\rm{D}}}^{{\rm{A}}1}$, ${K}_{{\rm{D}}}^{{\rm{B}}1}$ and ${K}_{{\rm{D}}}^{{\rm{A}}2}$, the value of [L] can be solved analytically with symbolic linear algebra software, such as Maxima (a Computer Algebra System, version 5.42.2, http://maxima.sourceforge.net). Once [L] is known, the concentrations of other species in the system are known from Eqs (28–33):

35 $$[{\rm{P}}]=\frac{\,{K}_{{\rm{D}}}^{{\rm{A}}1}\,{K}_{{\rm{D}}}^{{\rm{A}}2}\,{K}_{{\rm{D}}}^{{\rm{B}}1}[{{\rm{P}}}_{{\rm{total}}}]}{{K}_{{\rm{D}}}^{{\rm{A}}1}{[{\rm{L}}]}^{2}+({K}_{{\rm{D}}}^{{\rm{A}}2}\,{K}_{{\rm{D}}}^{{\rm{B}}1}+\,{K}_{{\rm{D}}}^{{\rm{A}}1}\,{K}_{{\rm{D}}}^{{\rm{A}}2})[{\rm{L}}]+{K}_{{\rm{D}}}^{{\rm{A}}1}\,{K}_{{\rm{D}}}^{{\rm{A}}2}\,{K}_{{\rm{D}}}^{{\rm{B}}1}}$$

36 $$[{\rm{PL}}]=\frac{[{\rm{P}}][{\rm{L}}]}{{K}_{{\rm{D}}}^{{\rm{A}}1}}$$

37 $$[{\rm{LP}}]=\frac{[{\rm{P}}][{\rm{L}}]}{{K}_{{\rm{D}}}^{{\rm{B}}1}}$$

38 $$[{\rm{LPL}}]=\frac{[{\rm{P}}]{[{\rm{L}}]}^{2}}{{K}_{{\rm{D}}}^{{\rm{B}}1}{K}_{{\rm{D}}}^{{\rm{A}}2}}$$

The lineshape analysis requires four more parameters to describe the 4-state system: the off rates for the four binding process: ${k}_{{\rm{off}}}^{{\rm{A1}}}$, ${k}_{{\rm{off}}}^{B1}$, ${k}_{{\rm{off}}}^{A2}$, and ${k}_{{\rm{off}}}^{{\rm{B2}}}$. The corresponding on rate is

39 $${k}_{{\rm{on}}}^{i}=\frac{{k}_{{\rm{off}}}^{i}}{{K}_{D}^{i}}$$

where *i* denotes any of the four binding steps. The predicted lineshape for a nucleus at a given titration point is described in Eqs (1–6). The following matrices are now

40 $${\bf{P}}=[\begin{array}{c}{[{\rm{P}}]}\\{[{\rm{PL}}]}\\ {[{\rm{LP}}]}\\{[{\rm{LPL}}]}\end{array}]$$

41 $${\bf{K}}=[\begin{array}{cccc}-({k}_{{\rm{o}}{\rm{n}}}^{{\rm{A}}1}+{k}_{{\rm{o}}{\rm{n}}}^{{\rm{B}}1})[{\rm{L}}] & {k}_{{\rm{o}}{\rm{f}}{\rm{f}}}^{{\rm{A}}1} & {k}_{{\rm{o}}{\rm{f}}{\rm{f}}}^{{\rm{B}}1} & 0\\ {k}_{{\rm{o}}{\rm{n}}}^{{\rm{A}}1}[{\rm{L}}] & -{k}_{{\rm{o}}{\rm{f}}{\rm{f}}}^{A1}-{k}_{{\rm{o}}{\rm{n}}}^{{\rm{B}}2}[{\rm{L}}] & 0 & {k}_{{\rm{o}}{\rm{f}}{\rm{f}}}^{{\rm{B}}2}\\ {k}_{{\rm{o}}{\rm{n}}}^{B1}[{\rm{L}}] & 0 & -{k}_{{\rm{o}}{\rm{f}}{\rm{f}}}^{{\rm{B}}1}-{k}_{{\rm{o}}{\rm{n}}}^{{\rm{A}}2}[{\rm{L}}] & {k}_{{\rm{o}}{\rm{f}}{\rm{f}}}^{A2}\\ 0 & {k}_{{\rm{o}}{\rm{n}}}^{{\rm{B}}2}[{\rm{L}}] & {k}_{{\rm{o}}{\rm{n}}}^{{\rm{A}}2}[{\rm{L}}] & -{k}_{{\rm{o}}{\rm{f}}{\rm{f}}}^{B2}-{k}_{{\rm{o}}{\rm{f}}{\rm{f}}}^{{\rm{A}}2}\end{array}]$$

### Fitting-specific algorithms

The fitting GUIs search the best-fit values of unknown thermodynamic and kinetic parameters based on the input lineshape data. The unknown parameters (e.g. *K*_D_, *k*_off_) are assigned to the initial values from user selection and then iteratively refined by an optimization algorithm to match the experimental data, such that the following target function (sum of squared errors between the experimentally determined and the predicted lineshape data) is minimized:

42 $$\sum _{i,j,k}{\{[{S}_{i,j}^{pred}({\omega }_{k}){a}_{i}+{b}_{i}]-{S}_{i,j}^{expt}({\omega }_{k})\}}^{2}$$

where ${S}_{i,j}^{pred}({\omega }_{k})$ and ${S}_{i,j}^{expt}({\omega }_{k})\,$are the predicted and experimental spectrum intensity of resonance *i* at titration point *j* at frequency ${\omega }_{k}$, respectively; *a*_*i*_ and *b*_*i*_ are the intensity and baseline correction factors for resonance *i* to compensate the normalization errors, if any, introduced during the normalization process of the experimental lineshape data.

If selected by the user in the interface, fitting errors of the best-fit values are determined by Monte Carlo resampling, in which a random noise is generated based on the spectrum noise level input with the experimental data and added to each of the input lineshape data points. The resampling and fitting procedure is repeated a minimum of 50 times and until convergence to determine the 95% confidence intervals of the fitted parameters. To determine experimental errors, multiple experiments should be performed and processed independently. The variance from experimental errors is generally larger than that from fitting errors^3^. If multiple experimental datasets are available, results from their independent fitting should be used to determine uncertainty for the fitted parameters.

## Supplementary information

Supplementary Figures and Table

## Data Availability

The datasets generated during and/or analyzed during the current study are available in the online tutorials from NmrLineGuru GitHub wiki (https://github.com/stonefonly/NmrLineGuru/wiki) or from the corresponding author.

## Code availability

The compiled GUIs and related documents are available from NmrLineGuru GitHub repo (https://github.com/stonefonly/NmrLineGuru). The software has been pre-installed in NMRbox (https://www.nmrbox.org). For academic use, this work should be cited. For commercial use or source code request, please contact us directly.
