# Supplementary material for: NmrLineGuru: Standalone and User-Friendly GUIs for Fast 1D NMR Lineshape Simulation and Analysis of Multi-State Equilibrium Binding Models
Source: Sci Rep. 2019 Nov 5;9:16023. doi: 10.1038/s41598-019-52451-8 (PMC6831641; doi:10.1038/s41598-019-52451-8)
Supplement: Supplementary file 1 — Supplementary Figures and Table [file 41598_2019_52451_MOESM1_ESM.pdf]

Supporting Information

NmrLineGuru: Standalone and User-Friendly GUIs for Fast 1D NMR Lineshape Simulation and Analysis of Multi-State Equilibrium Binding Models

Chao Feng[1], Evgenii L. Kovrigin[2], and Carol Beth Post[1,3,*]

[1]Department of Medicinal Chemistry and Molecular Pharmacology, Markey Center for Structural Biology, and Purdue Center for Cancer Research, Purdue University, West Lafayette, IN 47907, USA

[2]Magnetic Resonance Research Center, Department of Chemistry and Biochemistry, University of Notre Dame, Notre Dame, IN 46556, USA

[3]Department of Biological Sciences, Purdue University, West Lafayette, IN 47907, USA

*Correspondence to: Carol Beth Post, Department of Medicinal Chemistry and Molecular Pharmacology, Purdue University, West Lafayette, IN 47907, USA. E-mail: cbp@purdue.edu

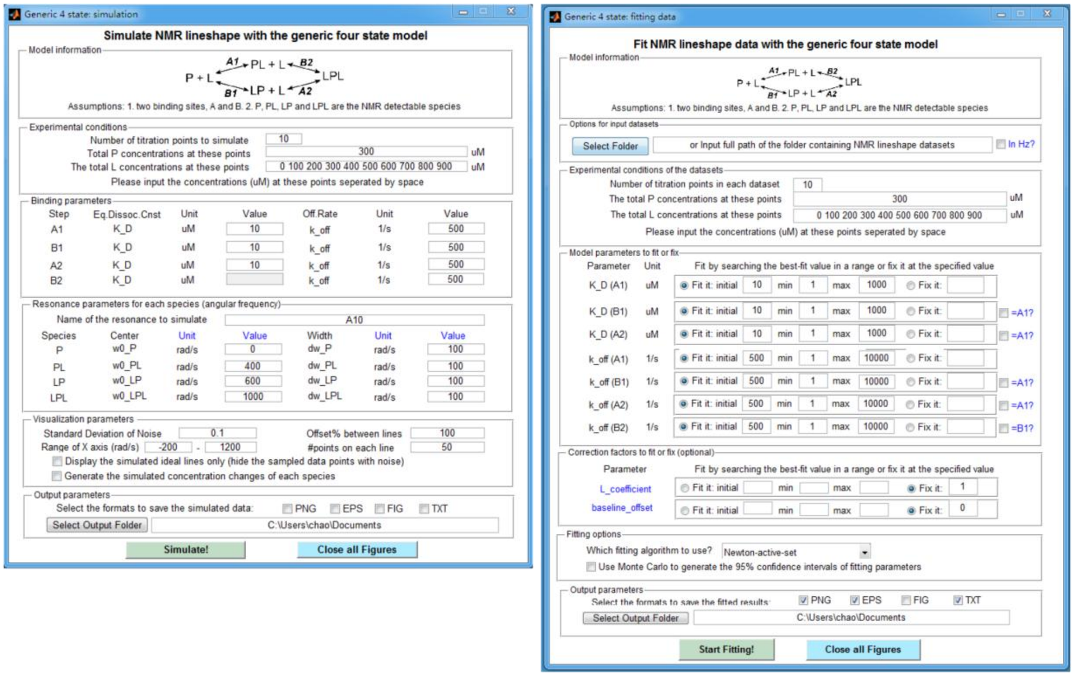

Figure S1. Lineshape simulation and fitting GUIs for the 4-state model. Each GUI is an independent single-window application. Example parameters are filled in and can be used for quick simulation or fitting. User-provided values enable customized simulations or fittings of NMR lineshape data.

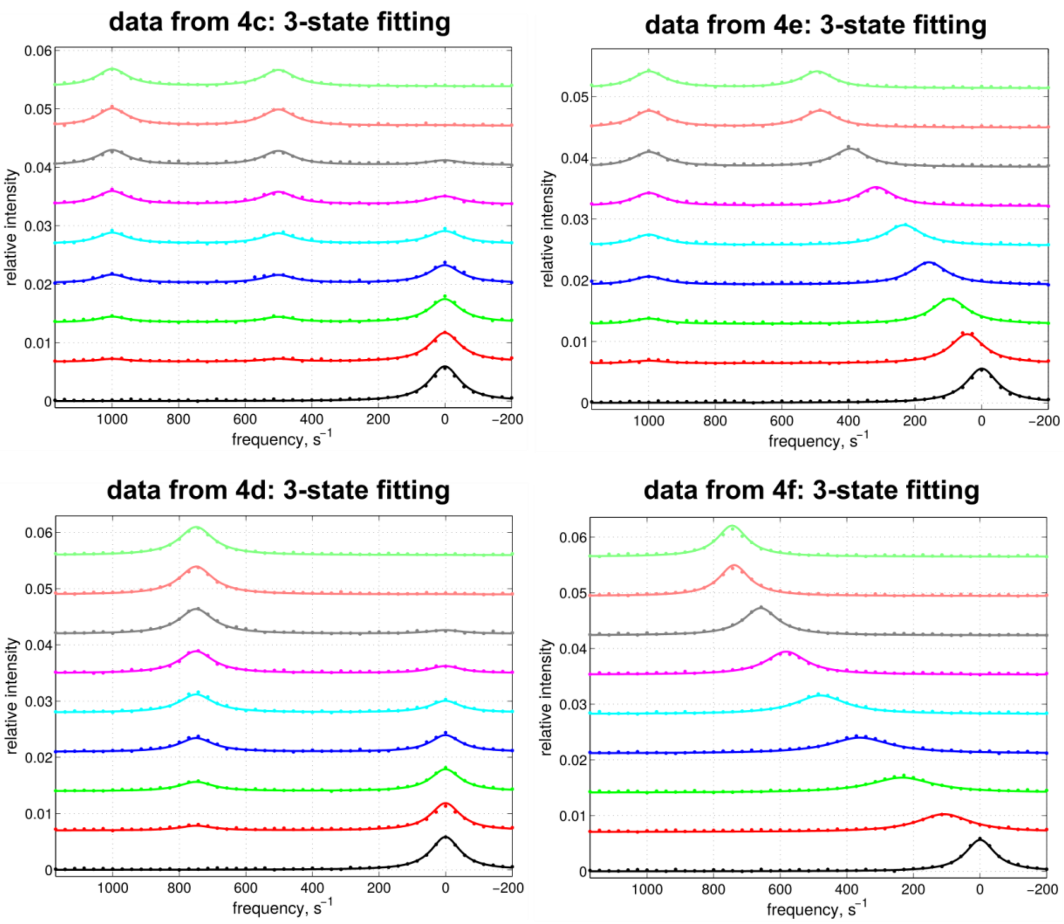

Figure S2. Fitting the simulated 3-state data with the 3-state model. The input data are from simulations shown in the main text Fig. 4c-f, as indicated. The spectra were simulated using either fast or slow kinetic rate constants for the first and second steps, with noise (signal/noise ≈ 50) and 50 data points on each line. The data are fitted with the 3-state fitting GUI. The sampled data are shown as dots and the fitted lineshape is shown as a smooth curve.

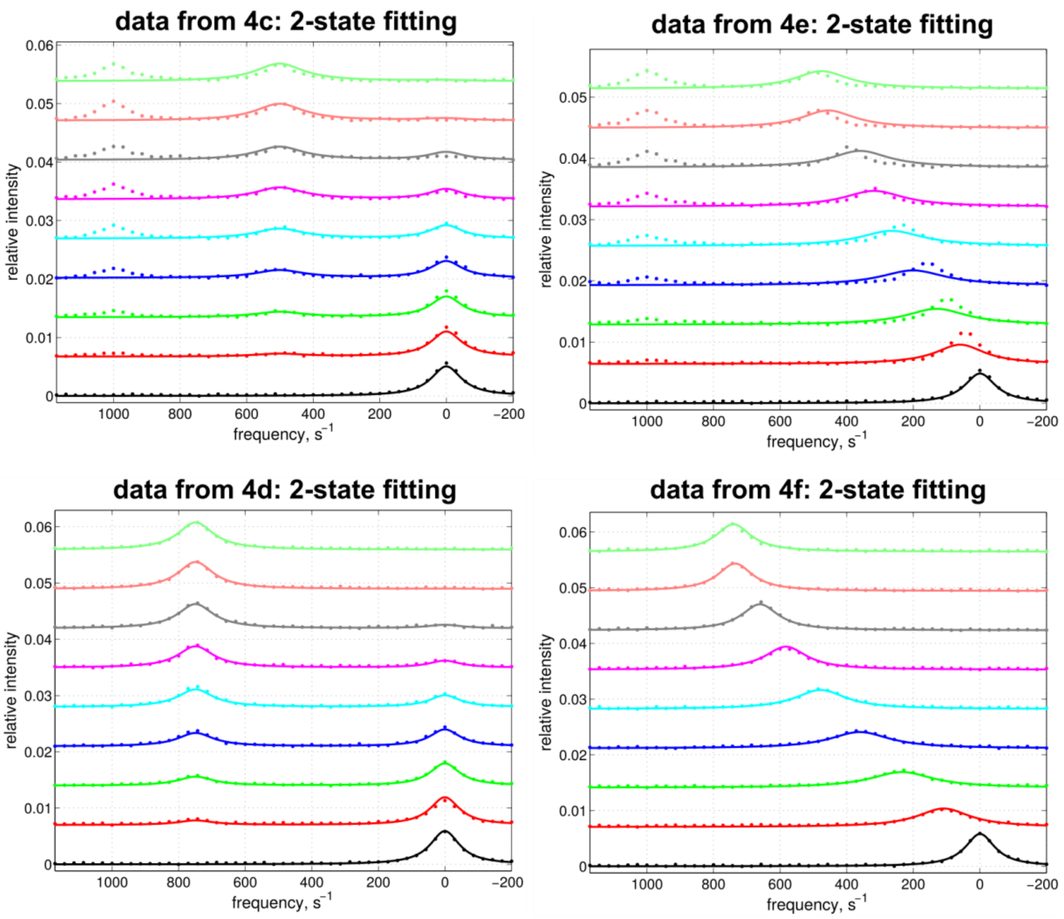

Figure S3. Fitting the simulated 3-state data with the 2-state model. The input data are described for Fig. S2. The data are fitted with the 2-state fitting GUI. The sampled data are shown as dots and the fitted lineshape is shown as a smooth curve.

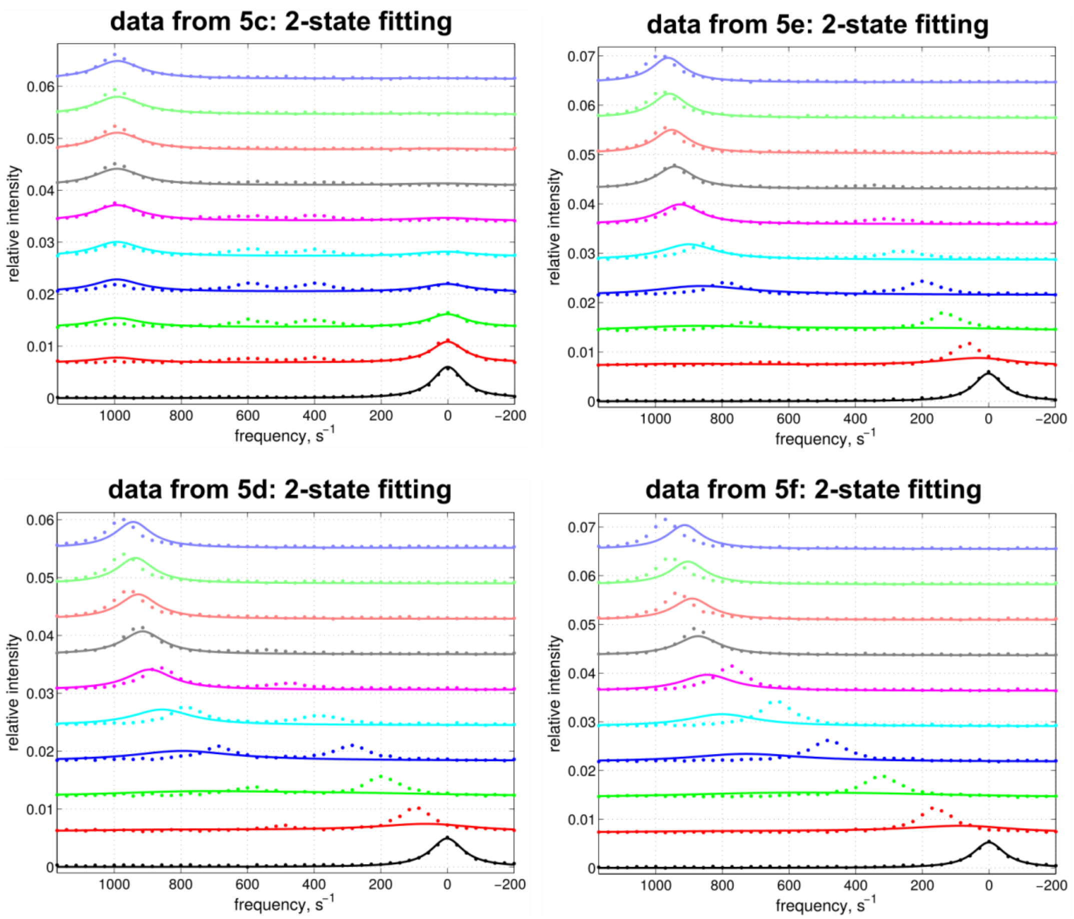

Figure S4. Fitting the simulated 4-state data with the 2-state model. The input data are from simulations shown in the main text Fig. 5 as indicated. The spectra were simulated using either fast or slow kinetic rate constants for the four binding steps, with noise (signal/noise ≈ 50) and 50 data points on each line. The data were fitted with the 2-state fitting GUI. The sampled data are shown as dots and the fitted lineshape is shown as a smooth curve.

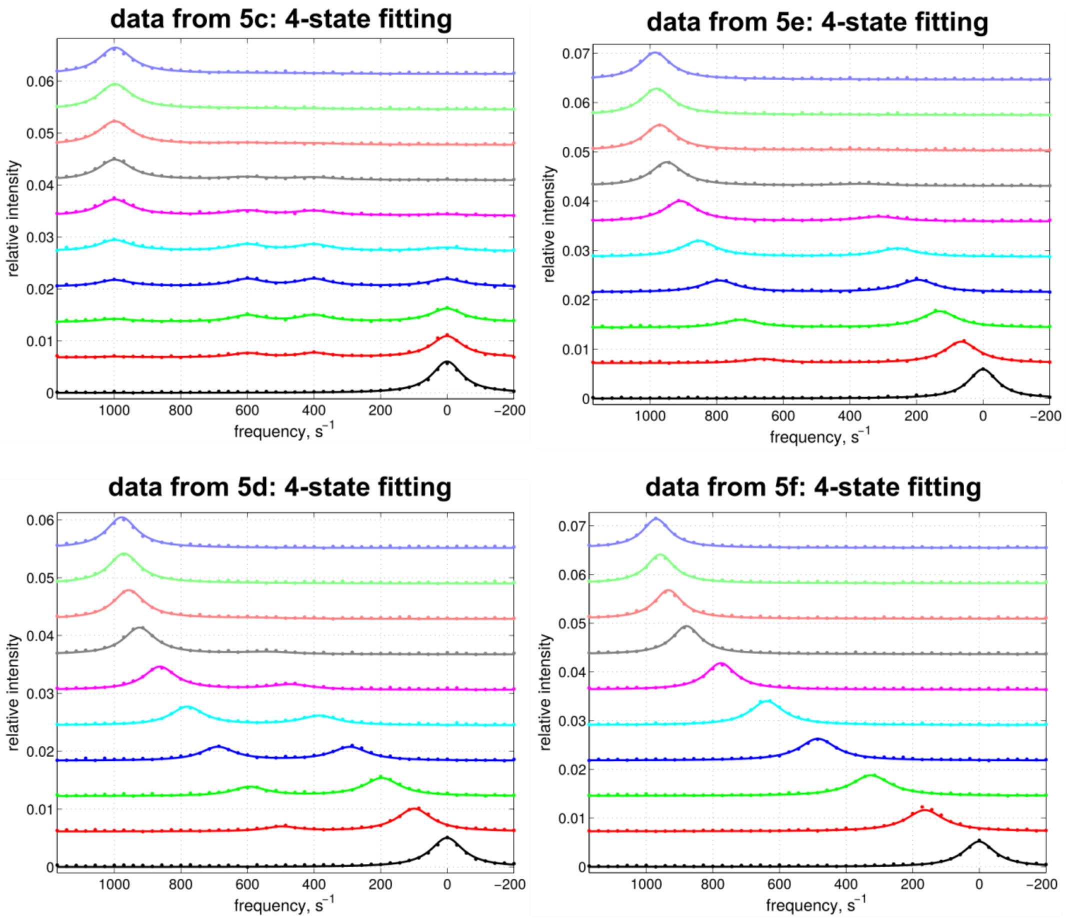

Figure S5. Fitting the simulated 4-state data with the 4-state model. The input data are described in Fig S4. The data were fitted with the 4-state fitting GUI. The sampled data are shown as dots and the fitted lineshape is shown as a smooth curve.
